# Identification of urine biomarkers associated with lung adenocarcinoma

**DOI:** 10.18632/oncotarget.15870

**Published:** 2017-03-03

**Authors:** Weiwei Wang, Shanshan Wang, Man Zhang

**Affiliations:** ^1^ Department of Pulmonary and Critical Care Medicine, Beijing Shijitan Hospital, Capital Medical University, Beijing, China; ^2^ Clinical Laboratory Medicine, Beijing Shijitan Hospital, Capital Medical University, Beijing, China; ^3^ Beijing Key Laboratory of Urinary Cellular Molecular Diagnostics, Beijing, China

**Keywords:** urine peptides, lung adenocarcinoma, MALDI-TOF MS, immunohistochemistry, biomarker

## Abstract

Lung adenocarcinoma (LAC) progression is accompanied by changes in protein levels that may be reflected in body fluids, such as urine. Urine collected from LAC patients (*n*=34) and healthy controls (*n*=36) was analyzed via matrix-assisted laser desorption/ionization time-of-flight mass spectrometry (MALDI-TOF-MS) combined with weak cationic exchange magnetic beads. The results revealed 76 urinary polypeptides significantly different between LAC patients and normal controls (*P*<0.05). Twenty-two of these peptides were up-regulated and 54 were down-regulated. Thirteen peptides had average peak intensities >600. Twelve of these 13 peptides were successfully identified using nano-liquid chromatography-tandem MS. Receiver operating characteristic analyses identified seven peptides with superior LAC diagnostic performances. Immunohistochemical staining in 20 paired LAC and adjacent normal tissues showed that IGKC, AAT, SH3BGRL3, osteopontin and gelsolin levels were higher in LAC tissues than in adjacent tissuesand were closely associated with LAC. Urinary peptides assessments may thus provide a novel, noninvasive, repeatable method for detecting and monitoring LAC. New, low-cost detection methods and bioinformatics tools are therefore urgently needed for the analysis of low abundance proteins and peptides in body fluids.

## INTRODUCTION

Lung cancer is one of most commonly diagnosed cancers and the leading cause of cancer death worldwide [1]. Lung adenocarcinoma (LAC) is the most common form of lung cancer, and constitutes nearly 40% of all lung cancer cases [2]. Lung cancer prognosis remains poor, and the 5-year survival rate of approximately 18% has not improved over several decades [1]. This is primarily due to late stage detection and a paucity of therapies effective against metastatic disease. Timely detection of lung cancer is hampered by several factors. First, there are no specific clinical symptoms in the early-stage of the disease. Second, aggressive therapeutic intervention is hampered by a relatively high median patient age [3]. Third, low-dose spiral computed tomography (LDCT) is unsuitable for widespread screening due to high costs and false positive rates. Moreover radiation exposure may increase the risk of lung cancer [4]. Finally, no accurate and reliable lung cancer biomarkers have been identified to date.

Protein expression and release differs between normal and cancer cells. Breakdown of large proteins often involves proteolytic processing, and progression to malignancy is often accompanied by changes in protease activities. Some cancer-specific peptides can be captured and analyzed to ascertain tumor status *in vivo*, and identification of such peptides might facilitate the development of biomarkers for early detection of lung cancer. Peripheral biofluids, such as serum and bronchoalveolar lavage fluid (BALF) have been used to detect lung cancer [5]. Recently, urine has attracted increased attention as a biospecimen because it remains relatively stable due to minimal post-sampling proteolysis. Urinalysis is non-invasive and urine is frequently accessible at large volumes without labor-intensive sample preparation. Urine markers may be clinically useful in diagnosing and monitoring bladder and prostate cancers [6–8]. Diagnostic tests based on urine polypeptides or protein markers can reportedly differentiate cholangiocarcinoma from benign biliary disorders, and may replace bile fluid analyses [9]. Tomasz *et al*. established a novel, three-protein biomarker panel to detect early-stage pancreatic cancer via urine [10]. Still, urine is seldom analyzed in the hunt for LAC biomarkers.

The peptidome, or the low-molecular-weight protein fragments and peptides in urine, represents an emerging tool for biomarker discovery. In recent years, mass spectrometry (MS) -based serum peptide screening has been used as a high-throughput approach to identify potential diagnostic and prognostic biomarkers for various diseases [11]. For instance, magnetic bead-based matrix assisted laser desorption/ionization time-of-flight mass spectrometry (MB-based MALDI-TOF-MS) technology was used to identify serum peptide fingerprints in esophageal squamous cell carcinoma [12]. Detection of low abundance proteins is possible, and quantitative information can be obtained from the spectral counts. However, urine peptidome studies in lung cancer have not simultaneously evaluated candidate biomarkers in patient tumor tissue [13].

In this study, we detected proteomic changes in urine samples from 70 LAC and control samples. Urine peptides were purified using weak cation-exchange magnetic beads (WCX-MB),. MALDI-TOF-MS was used to analyze peptide expression profiles. Candidate diagnostic peptides were then identified by nanoliquid chromatography-tandem MS,. and were further verified using immunohistochemical staining in tissue sample sets. Our results indicated that specific urinary peptides were closely associated with LAC. The identified peptides might serve as potential biomarkers to noninvasively detect LAC, through analysis of urine samples.

## RESULTS

### Urinary peptidome profiling

**Figure 1:**
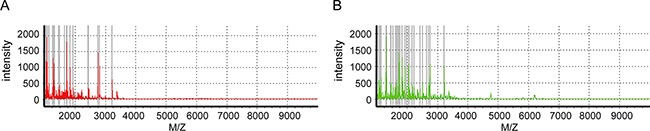
Urine peptide fingerprints in the range of 1000-10,000 m/z Mass spectra of **A**. lung adenocarcinoma (red, n =23) and **B**. normal controls (green, n =16) created by ClinTOF.

Urine samples from 70 volunteers were purified using magnetic beads and exhibited spectral peaks in the range of 1000–10,000 Da. Typical WCX representative spectra for LAC and normal control patient samples following MALDI-TOF MS analysis are shown in Figure 1. Peak position and peak intensity differences were observed between the two groups.

### Peptide screening

**Figure 2:**
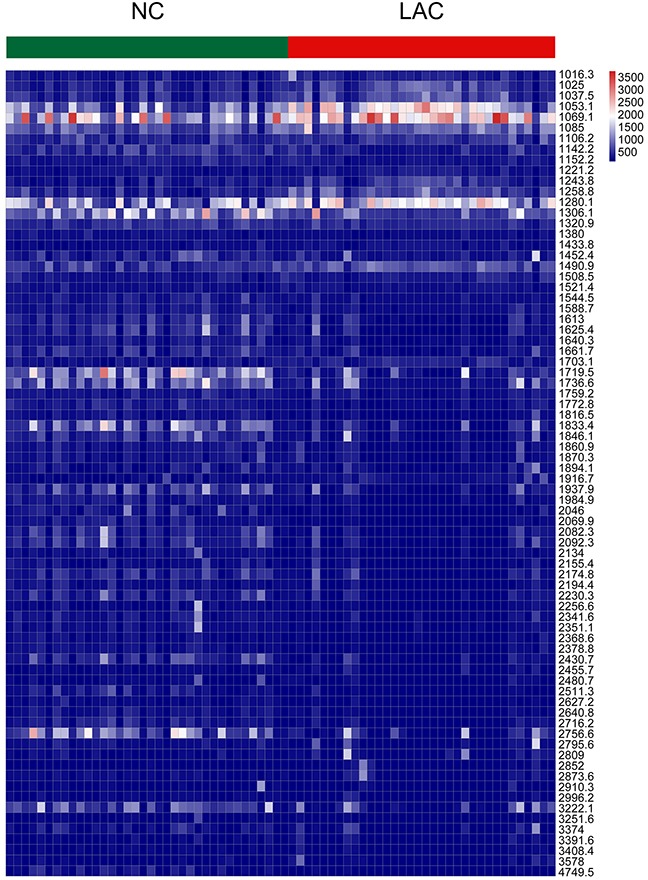
Columns represented samples, rows were m/z peaks (in numerical order), the heat map scales of peak intensities range from blue to red with the transitional midpoint on white

**Figure 3:**
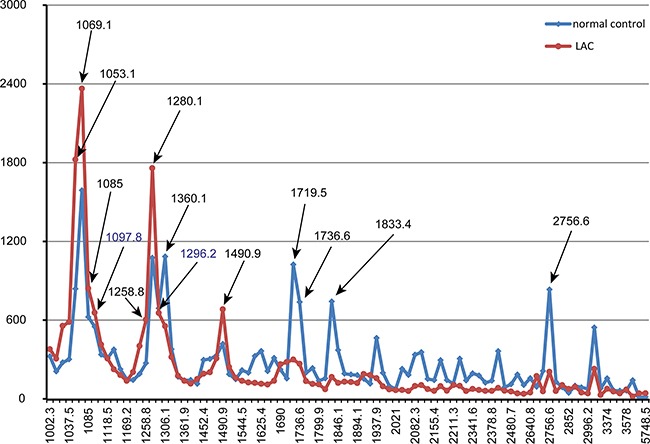
The distribution of average peak area from two groups and arrows indicated peaks that average intensity was higher than 600 in LAC group or normal control

**Figure 4:**
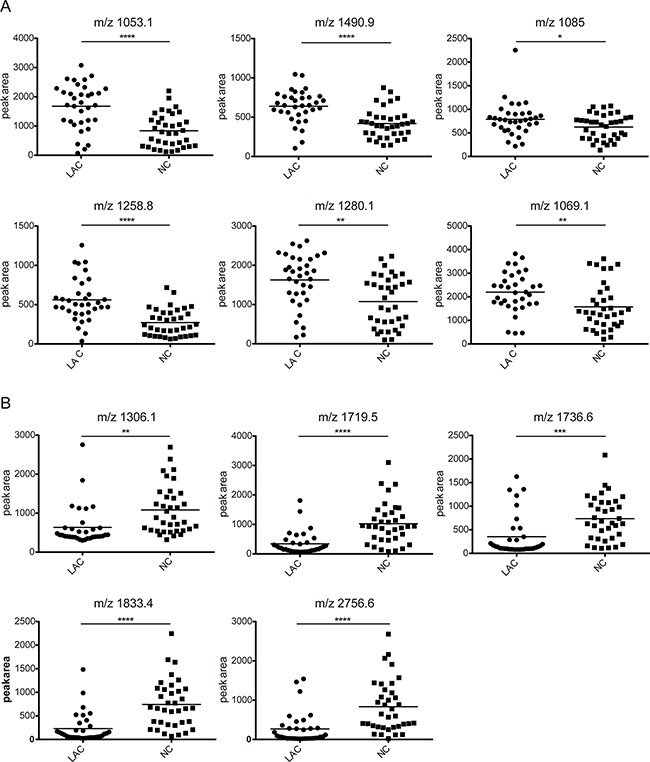
The feature of the 11 selected peaks in lung adenocarcinoma and health control The peak area distributions in all samples. **A**. The average value showing an increasing trend in lung adenocarcinoma group compared with normal control group. **B**. The average value showing a decreasing trend in lung adenocarcinoma group compared with normal control group. *P<0.05, **P<0.01, ***P<0.001, ****P<0.0001.

MALDI-TOF analysis detected a total of 94 peaks with m/z spectra ranging from 1000 to 10,000 Da. Seventy-six out of 94 features differed between LAC patients and normal controls (*P*<0.05) (Figure 2). Among these, 22 peptides were upregulated and 54 were downregulated in LAC patients compared to controls. Thirteen peptide peaks had average intensities > 600 in the LAC or normal control groups. The mass-to-charge ratios of these peaks were: 1053.1, 1490.9, 1280.1, 1085, 1258.8, 1069.1, 1306.1, 1719.5, 1736.6, 1833.4, 2756.6, 1097.8, and 1296.2 (Figure 3). Compared to normal control, six peptides (m/z 1053.1, 1490.9, 1280.1, 1085, 1258.8, and 1069.1) were upregulated in LAC patients (Figure 4A, Table 1) while five (m/z 1306.1, 1719.5, 1736.6, 1833.4, and 2756.6) were downregulated (Figure 4B, Table 1). Two peptides (m/z 1097.8 and 1296.2) were the same between LAC patients and normal controls (Table 1, *P*>0.05).

**Table 1:** The characteristic of thirteen selected peaks

Peptides peaks(m/z)	*P* value	Cancer group	Control group
1053.1	<0.0001	1679.8±772.5	839.0±564.7
1490.9	<0.0001	639.4±205.7	418.9±187.9
1085	0.0430	789.4±356.7	623.1±263.3
1258.8	<0.0001	560.5±273.8	271.6±171.2
1280.1	0.0010	1625.4±666.8	1075.3±646.1
1069.1	0.0080	2196.6±856.3	1588.5±983.0
1306.1	0.0010	637.2±503.1	1083.9±620.7
1719.5	<0.0001	343.2±390.7	1023.7±700.8
1736.6	0.0010	352.3±439.5	737.1±462.4
1833.4	<0.000	230.7±315.4	742.0±508.0
2756.6	<0.000	269.5±401.6	832.6±662.1
1097.8	0.2720	616.3±248.2	550.0±252.2
1296.2	0.7530	663.9±293.2	689.5±378.0

The peak area of every peak in two groups was presented as mean ± SD. *P* value was calculated by *t*-test (normally distributed continuous data) or Mann-Whiney *U*-test (non-normally distributed continuous data). *P*<0.05 was considered as statistically significant difference.

### ROC analysis

**Figure 5:**
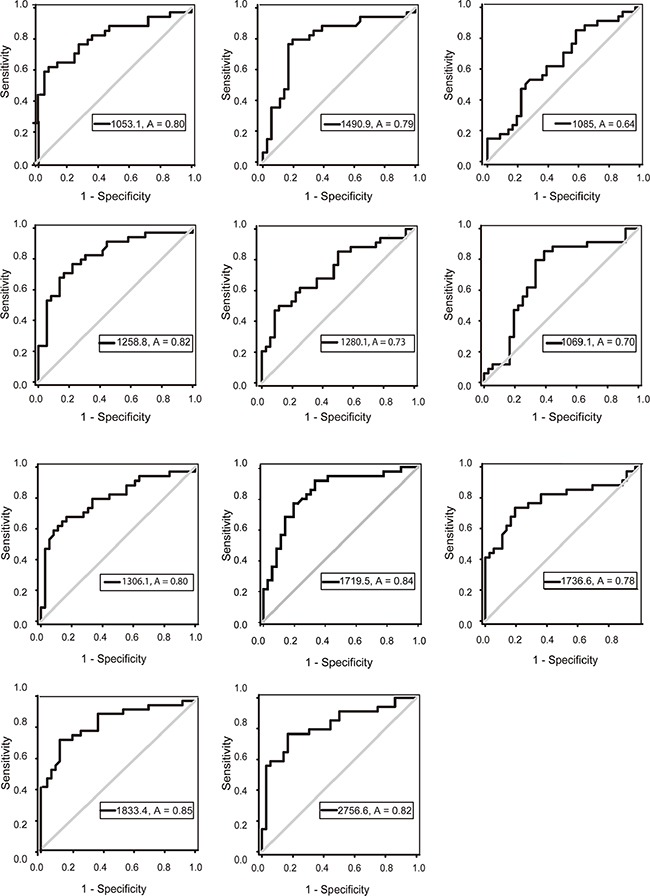
ROC curves of potential urine biomarker levels for differentiating LAC from the normal control The AUCs of m/z 1053.1, 1258.8, 1490.9, 1306.1, 1719.5, 1833.4, 2756.6 were 0.81, 0.83, 0.80, 0.80, 0.84, 0.86, 0.82. The AUCs of m/z 1069.1, 1085, 1280.1, 1736.6 were 0.70, 0.64, 0.73, 0.79, respectively.

For the thirteen peptides with average peak intensities >600, ROC analyses were performed to calculate the sensitivities, specificities and accuracies at different cut-off points. For the ROC curves, the AUCs of m/z 1053.1, 1258.8 and 1490.9 were 0.81 (95% CI 0.67-0.91; *P*<0.0001), 0.83 (95% CI 0.73-0.92; *P*<0.0001) and 0.80 (95% CI 0.68-0.90; *P*<0.0001), respectively. Sensitivities and specificities were 62% and 92% for m/z 1053.1, 77% and 78% for m/z 1258.8, and 79% and 81% for m/z 1490.9. These three peptides were upregulated in the LAC group. The AUCs of m/z 1306.1, 1719.5, 1833.4 and 2756.6 were 0.80 (95% CI 0.69-0.90; *P*<0.0001), 0.84 (95% CI 0.74-0.93; *P*<0.0001), 0.86 (95% CI 0.77-0.94; *P*<0.0001), and 0.82 (95% CI 0.72-0.92; *P*<0.0001), respectively. Sensitivities and specificities were 68% and 83% for m/z 1306.1, 91% and 67% for m/z 1719.5, 74% and 89% for m/z 1833.4, and 77% and 83% for m/z 2756.6. These four peptides were downregulated in the LAC group. Peptides with m/z 1280.1, 1069.1, 1085, and 1736.6 had limited clinical utility, with AUCs of 0.73 (95% CI 0.61-0.85; *P*=0.001), 0.70 (95% CI 0.57-0.83; *P*=0.004), 0.64 (95% CI 0.51-0.77; *P*=0.043) and 0.79 (95% CI 0.67-0.90; *P*<0.0001), respectively. Sensitivities and specificities were 50% and 89% for m/z 1280.1, 85% and 61% for m/z 1069.1, 85% and 42% for m/z1085, and 74% and 81% for m/z 1736.6. Peptides with m/z 1280.1, 1069.1, and 1085 were upregulated in the LAC group, while the peptide with m/z 1736.6 was downregulated (Figure 5).

### Biomarkers identification

Peptides sequencing via nano-liquid chromatography-tandem MS successfully identified 12 out of 13 peaks with average intensities >600. The Mascot search of the Uniprot-SwissProt Human database provided protein name. The m/z 1069.1 peak sequence was not identified. Detailed identification results are shown in Table 2.

**Table 2:** Identified peptides sequence of the selected peaks

Swiss-ProtAcc. No.	m/z	Molecularweight	peptide sequences	Protein name
A0A087×130	1053.1	1052.5	LNNFYPRE	Ig kappa chain C region(IGKC)
P01009	1490.9	1494.7	FGDTEEAKKQIND	Alpha-1-antitrypsin
P01625	1280.1	1276.6	DIVMTQSPDSLA	Kappa chain V-IV region Len
Q5T123	1085	1086.5	YSTSVTGSRE	SH3 domain-binding glutamic acid-fat-like protein 3 (SH3BGRL3)
A0A087×1V9	1258.8	1258.7	YLQKPGQSPQL	Protein IGKV2-28
P01009	1306.1	1307.7	MIEQNTKSPLF	Alpha-1-antitrypsin
Q5T123	1719.5	1719.9	LAGNPKATPPQIVNGDQ	SH3 domain-binding glutamic acid-rich-like protein 3 (SH3BGRL3)
P01040	1736.6	1736.9	IPGGLSEAKPATPEIQE	Cystatin-A
P10451	1833.4	1833.9	ESEELNGAYKAIPVAQD	Osteopontin
Q5T0H9	2756.6	2759.4	TAQLDEELGGTPVQSRVVQGKEPAHL	Gelsolin
Q5T0H9	1097.8	1096.6	FVLKTPSAAY	Gelsolin
D6RBV2	1296.2	1297.5	LDTYPNDETTE	Vesicular integral-membrane protein VIP3(LMAN2)
	1069.1			Identification failure

### IGKC, AAT, SH3BGRL3, osteopontin, gelsolin, cystatin-A and LMAN2 expression in LAC

**Figure 6:**
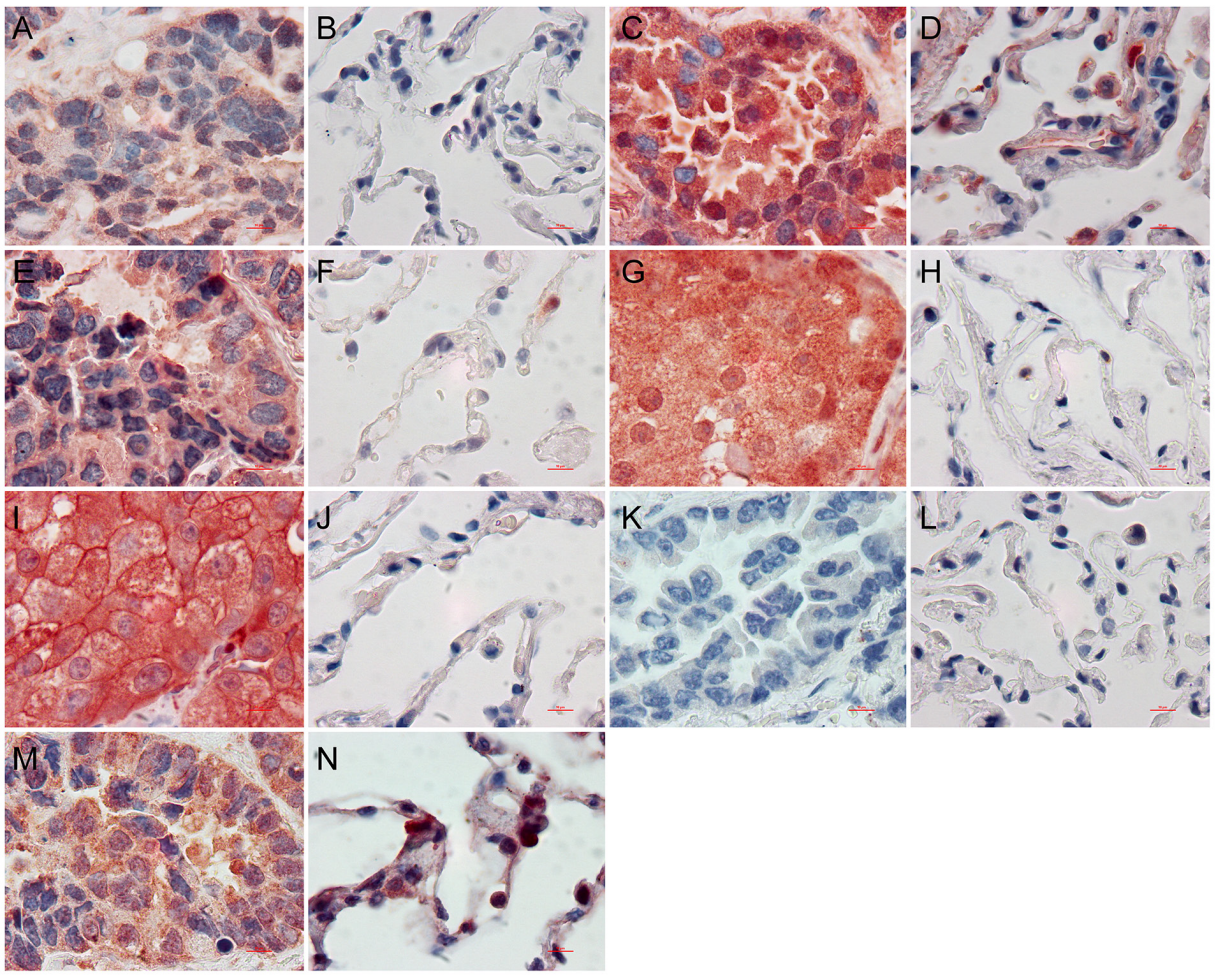
The expression of proteins using immunohistochemistry assay IGKC **A**. AAT **C**. SH3BGRL3 **E**. osteopontin **G**. gelsolin **I**. in LAC tissues were significantly different with IGKC **B**. AAT **D**. SH3BGRL3 **F**. osteopontin **H**. gelsolin **J**. in normal lung tissues, respectively. Cystain-A was negative in LAC **K**. and adjacent normal lung tissue **L**. LMAN2 was positive in LAC **M**. and adjacent normal lung tissue **N**. (×1000).

Immunohistochemistry was employed to assess IGKC, AAT, SH3BGRL3, osteopontin, gelsolin, cystatin-A and LMAN2 levels in 20 paired LAC and adjacent normal tissues. Staining was primarily observed in the cytoplasm. IGKC, AAT, SH3BGRL3, osteopontin and gelsolin levels were increased in LAC tissues compared to controls (*P*<0.05; Figure 6, Table 3). There were no cystain-A or LMAN2 expression differences between LAC tissues and controls (*P*>0.05).

**Table 3:** The expressions of IGKC, AAT, SH3BGRL3, osteopontin, gelsolin, cystain-A and LMAN2 in LACs and adjacent normal lung tissues

Proteins	LAC(*n*=20)		Normal lung tissue(*n*=20)		*P*
	4-12	0-3	4-12	0-3	
IGKC	5	15	0	20	0.047
AAT	7	13	1	19	0.044
SH3BGRL3	9	11	2	18	0.031
osteopontin	7	13	1	19	0.044
gelsolin	7	13	1	19	0.044
cystain-A	2	18	0	20	0.487
LMAN2	9	11	3	17	0.273

## DISCUSSION

Urine as an analytical body fluid has several advantages over blood. Urine collection is noninvasive and can be repeated to obtain sufficient sample quantities at multiple time points. Urine proteins are also relatively stable due to low levels of proteolytic degradation. Furthermore, urine harbors lower numbers of polypeptides and proteins as compared to other body fluids, and has a lower dynamic range of protein concentrations. Blood, on the other hand, contains 20 kinds of high abundance proteins which correspond to 99% of the proteins in the sample; these high abundance proteins mask other less abundant, potentially clinically usefulproteins [14].

More than 2,300 different proteins have been detected in urine [15]. Proteomic analyses of urine suggest that it contains information specific to a number of kidney diseases, as well as cardiovascular and brain diseases, and certain types of cancer [16]. Here, we successfully established a peptide marker panel based on urinary peptides that appear to reflect LAC progression.

We used WCX-MB coupled with MALDI-TOF-MS to analyze human urine peptidomes, and distinguished LAC-specific proteinaceous biomarkers present in urine. We successfully identified and validated a portion of these biomarkers, and developed a urine-based LAC diagnostic test. To the best of our knowledge, this was the first assessment of potential LAC biomarkers in human urine using WCX coupled with MALDI-TOF-MS. These biomarkers might be used to monitor patients at high risk for LAC. Seventy-six m/z peaks differed between LAC patients and normal controls. Six of these peaks (m/z 1053.1, 1490.9, 1280.1, 1085, 1258.8 and 1069.1) were upregulated in LAC with peak intensities > 600. These peptides were identified as Ig kappa chain C region (IGKC), alpha-1-antitrypsin (AAT), IGKV2-28, Ig kappa chain V-IV region Len, SH3 domain-binding glutamic acid-rich-like protein 3 (SH3BGRL3). We hypothesized that upregulated proteins in lung cancer patient urine are most likely derived from cancer cells. Immunohistochemistry results in 20 surgically resected LACs and adjacent normal tissues showed that IGKC, AAT and SH3BGRL3 were more highly expressed in tumor cells as compared to non-tumor tissues. These proteins might play a role inLAC development.

Five peptides (m/z 1306.1, 1719.5, 1736.6, 1833.4 and 2756.6) were down-regulated in LAC patients, with peak intensities >600 in normal controls. The downregulated peptide, m/z 1306.1, and the upregulated peptide, m/z 1490.9, were different fragments of AAT. The downregulated peptide, m/z 1719.5, and the upregulated peptide, m/z 1085, were different fragments of SH3BGRL3. The downregulated peptide, m/z 2756.6, and the peptide, m/z 1097.8, were different fragments of gelsolin. The surprising finding that different fragments of the same protein were differentially expressed in LAC vs normal tissuess will be explored in future work.

The peaks, m/z 1736.6 and 1833.4, were identified as cystatin-A and osteopontin. Consistent with previous studies, gelsolin and osteopontin exhibited higher expression in LAC tissues than adjacent normal tissues [17–19]. These proteins were elevated in blood and play important roles in tumor progression and metastasis [20, 21], but both were downregulated in LAC patient urine samples. This indicated that the formation of urine peptides is a complex process that necessitates further study.

We found, unexpectedly, that LAC patients urine samples contain elevated levels of some immunoglobulin (Ig) light chains, such as IGKC, Ig kappa chain V-IV region Len, protein IGKV2-28 and Ig lambda-2 chain C regions. It is generally accepted that under normal conditions, mature B lymphocytes are the sole source of immunoglobulins. This elevated antibody levels are likely induced by a disordered immune system. Based on our findings, IGKC may arise from cancer cells themselves. Previous studies confirmed Ig expression in many non-hematopoietic cancer cells, including breast, colon, lung, liver, cervical and oral cancers. Human epithelial cancer produces IgG in both cytoplasmic and secreted forms [22]. Xiao, *et al*. detected the Ig kappa V and Ig alpha C regions in serum-free conditioned medium from primary lung cancer cells [23]. Cancer-associated Ig shared some characteristics with normal Ig produced by B lymphocytes, but differed with respect to genetic processing [24, 25], transcription [26], expression [25], protein structure [27], post-translational modification [27] and biological function [28–31]. These results suggested that aberrant Ig-like molecule expression might represent a common feature of malignant epithelial cells. These Ig-like molecules are reflected in blood and urine and may contribute to cancer occurrence and progression. IGKC, Ig kappa chain V-IV region Len, protein IGKV2-28, and Ig lambda-2 chain C regions have not, to our knowledge, been previously associated with lung cancer.

AAT is a serine protease inhibitor synthesized primarily in liver, but also in extra-hepatic tissues and cells, including tumor cells. AAT overexpression has been observed in patients with various tumors, including lung cancer [32, 33]. Plasma AAT levels are reportedly elevated in lung cancer patients [34–36]. AAT is required for cancer cell migration, invasion, and pericellular fibronectin assembly [37]. The C-terminal fragment of AAT induced tumor cell proliferation and invasiveness in human pancreatic adenocarcinoma [38], melanoma [39] and breast carcinoma cells [40]. In contrast, AAT downregulation by short hairpin RNA (shRNA) suppressed cell proliferation, metastasis, and adhesion in human lung adenocarcinoma A549 cells and in the lung tissues of a K-rasLA1 mouse model [32]. On the other hand, AAT deficiency may increase lung cancer risk [41]. Still, AAT-positive adenocarcinomas are associated with worse prognoses as compared to AAT-negative tumors [33]. Our results demonstrated that AAT peptides in LAC patients urines differed from those in normal controls. The AAT protein was elevated in lung cancer tissues, suggesting that AAT might play promote carcinogenesis *in vivo*, in addition to its role as proteinase inhibitor. Importantly, AAT peptides in urine might be potential LAC biomarkers.

SH3BGRL3 is a member of the SH3BGR protein family, but lacks the typical SH3- and Homer EVH1-binding motifs, suggesting a function different from that of other subfamily members [42]. SH3BGRL3 was upregulated in glioblastoma multiformand primary bladder cancers as compared to non-tumor tissues, and was detected inurothelial carcinoma patient urine [43, 44]. SH3BGRL3 appears to promote cancer cell proliferation, epithelial-mesenchymal transition (EMT), and cell migration. We found that SH3BGRL3 interacts with epidermal growth factor receptor (EGFR) at Y1068, Y1086, and Y1173 through Grb2 via its proline-rich motif,and activates the Akt-associated signaling pathway [44]. This protein had not been previously associated with lung cancer. Our results demonstrated that SH3BGRL3 peptides distinguished LAC patient urine from normal control samples.

In conclusion, our study identified urine peptides closely associated with LAC, and measurement of these peptides in urine using WCX-MALDI-TOF may specifically detect LAC. These biomarkers must be validated in larger numbers of urine samples from additional lung cancer patients. At present, the biomarkers identified here have not been applied clinically due in part to the high costs and statistical processing requirements of our methods. Therefore, new detection methods and bioinformatics tools are urgently needed for the analysis of low abundance proteins and peptides from body fluids.

## MATERIALS AND METHODS

### Study subjects

This research was approved by the ethics committee of Beijing Shijitan Hospital, Capital Medical University (research ethics review No. 5, 2014). Patients with LAC and healthy individuals were recruited from Beijing Shijitan Hospital from October 2014 until December 2015, following a clinical check of renal function and urinary sediment at the same medical facility. All parcitipants provided written informed consent in accordance with the provisions of the Helsinki Declaration. All LAC patients were pathologically diagnosed by two senior pathologists. Thirty-six healthy individuals were recruited from a healthy public population cohort (Beijing, China) undergoing annual medical examination in Beijing Shijitan Hospital. LAC patients and healthy individuals characteristics are provided in Table 4. No urine samples exhibited hematuresis and urinary albumin/creatinine ratios (A/Cr) were < 30mg/g. We also utilized 20 LAC and adjacent nontumor tissue pairs, of which 20 were a subset of the tissue set. Adjacent normal tissues were at least 5 cm distant from the tumor. Individuals excluded from the study if they had received preoperative chemotherapy or radiotherapy. LAC patients were separated according to the 2009 TNM classification of malignant tumors by the International Union Against Cancer and the American Joint Committee on Cancer.

**Table 4:** Demographics of patients and normal control subjects

	Urine set		Tissue set	
	Cancerpatient(*n*=34)	Normalcontrol(*n*=36)	*P*	Tumor/adjacentnormal pairs(*n*=20)
**Age**	62.6±11.1	62.3±10.6	*P*=0.52	65.4±9.8
**Gender**				
Female	14(41%)	15(42%)	*P*=0.98	8(40%)
Male	20(59%)	21(58%)		12(60%)
**Smoking habit**				
Nonsmoker	22(65%)	23(64%)	*P*=0.94	12(60%)
Ever smoker	12(35%)	13(36%)		8(40%)
**Clinical stage**				
I-II	14			8
III-IV	20			12

### Urine collection

Midstream urine samples (50 ml) were collected by sterile polypropylene tubes in the morning and were immediately centrifuged at 400 g for 15 min to remove cell debris and casts. Supernatants were divided into aliquots and stored at −80°C.

### Fractionation of urine peptides

All samples were fractionated by WCX-MB according to the manufacture's instructions (Bruker Daltonics). Samples were purified and separated through binding, washing and elution. First, 10 μl MB-WCX, 95 μl WCX-MB binding solution and 10 μl urine were mixed thoroughly in a polypropylene tube, and then incubated for 5 min. Tubes were placed in the magnetic bead separation device (Bruker Daltonics) for 1 min to separate the unboud solution. Magnetic beads were then washed three times with 100 μl magnetic bead washing solution. Second, 10 μl MB-WCX elution solution was added to the beads and mixed by vortexing. Finally, the supernatant was transferred into a fresh tube. 5 μl MB-WCX stabilizing solution was added. The well-mixed eluate was then stored at −20°C.

### MALDI-TOF-MS and data processing

Urine sample eluates were diluted 1:10 in matrix solution containing α-cyano-4-hydroxycinnamic acid (Bruker Daltonics). Then, 1 μl of the resulting mixture was spotted onto the AnchorChip target (Bruker Daltonics), allowed to air dry, and ionized by a nitrogen laser (λ=337 nm) operating at 25 Hz. MALDI-TOF MS was performed using an Autoflex TOF instrument (Bruker Daltonics). Three standard peptides were used as an external standard preparation to ensure the average molecular weight deviation was no more than 100 ppm. For data processing, all spectra obtained from urine samples were analyzed using BioExplorer™ software (Bioyong Tech, Beijing, China). Each spectrum was normalized using total ion count. Peak m/z values or intensities in the 1000-10000 Da range from all signals with a signal-to-noise ratio >5 were determined. To align the spectra, a mass shift of no more than 0.1% was determined. Peaks that were detected in >80% of samples were considered informative. The t test was applied to compare the peak volumes in the two groups. Peaks with adjusted *p* values < 0.05 and average peak intensities >300 were regarded as statistically significant. Peak area was used as quantitative standardization. Thenceforth, the RBF algorithm was used to find the best pattern for distinguishing LAC.

### Peptide biomarkers identifications

Identification of differentially expressed peptides sequences was performed using a nano-LC/ESI-MS/MS system consisting of an Aquity Ultra Performance LC (UPLC) system (Waters, USA) and an LTQ Orbitrap MS(Thermo Scientific, Germany) equipped with a nano-ESI source. Desalted peptides were analyzed using a C18 analytical column (75 um×500 mm, 100 Å Magic, 2 μm) at a flow rate of 300 nl/min with the mobile phases A (5% acetonitrile, 0.1% formic acid, Sigma-Aldrich, USA) and B (95% acetonitrile, 0.1% formic acid). The gradient elution profile was as follows: 5%B-5%B-30%B-90%B-5%B over 60 min. The MS instrument was operated in a data-dependent model. Proteome Discoverer (PD) version 1.4 (Thermo -Scientific) was used to search against with the Uniprot-SwissProt Human protein database for the raw data files. The search engine Mascot (version 2.3.2) was implemented in PD as per the manufacturer's instructions. Downloaded files were searched directly using Mascot through PD. The search parameters were as follows: 50 ppm tolerance for precursor ion masses, 0.8 Da for fragment ion mass tolerance, a false discovery rate (FDR) ance,no enzymes, no fixed modification and variable modification.

### Immunohistochemistry

LAC and adjacent non-tumor tissues obtained during surgery were fixed in 10% formalin, embedded in paraffin, and sectioned into 4-mm slices. Slides were deparaffinized for 20 minutes in xylene and then dehydrated in 100%, 100%, 95%, and 75% alcohol, for 2 minutes at each concentration. After five 10-min rinses in phosphate-buffered saline (PBS), antigen retrieval was performed by heating slides in a pressure cooker with antigen unmasking solution. Slides were then washed with PBS for 10 min, incubated for 15 min in 3% H_2_O_2_, and rinsed again with PBS for 10 min. After antigen retrieval, samples were incubated at 4°C overnight with antibodies respectively (the antibodies were listed in Table 5). Samples were then washed with PBS, incubated with horseradish peroxidase-conjugated secondary antibody (Beijing Zhongshan Jinqiao Biotechnology, Beijing, China) for 20 min at 37°C and then washed again with PBS for 15 min. Samples were stained using chromogen 3,3′-diaminobenzidine solution (Beijing Zhongshan Jinqiao Biotechnology, China) for 5 min, counterstained with hematoxylin for 2 min, dehydrated with 75%, 95%, 100%, and 100% alcohol, cleaned with xylene, and sealed with natural gum.

**Table 5:** The characteristic of antibodies

Antibody name	Species reactivity	Clonality	dilution	manufacturers
anti-Ig kappa chain C region (IGKC) antibody	mouse anti-human	monoclonal	1:200	Abcam, UK
anti-alpha 1 antitrypsin antibody	mouse anti-human	monoclonal	1:300	Abcam, UK
anti-SH3 domain-binding glutamic acid-fat-like protein 3 (SH3BGRL3) antibody	rabbit anti-human	polyclonal	1:100	Abcam, UK
anti-osteopontin antibody	mouse anti-human	monoclonal	1:300	Abcam, UK
anti-cystain A antibody	mouse anti-human	monoclonal	1:50	Abcam, UK
anti-gelsolin antibody	rabbit anti-human	monoclonal	1:400	Abcam, UK
anti-LMAN2 antibody	rabbit anti-human	polyclonal	1:100	Abcam, UK

Immunostaining was blindly evaluated by two independent experienced pathologists using a light microscope (Nikon Ci-S, Japan). Images were captured using NIS Elements F software (Nikon). The scoring method was described previously [45], Using at least 10 randomly selected high-power fields. Each specimen was scored according to staining intensity (intensity) and area (extent). Staining intensity was scored as follows: “0”, no staining; “1”, mild staining; “2”, moderate staining and “3”, intense staining. The percentage of positive cells was divided into five categories, “0”, no staining; “1”, 1–10%; “2”, 11–50%; “3”, 51–80%; “4”, 81–100%. Staining intensity and percentage were multiplied to produce a total score. A total score of 4–12 was defined as positive expression, and 0–3 was considered negative.

### Statistical analysis

SPSS software 22.0 was used to calculate all statistical comparisons. A *t*-test (normally distributed continuous data) or Mann-Whiney U test (non-normally distributed continuous data) was employed to compare polypeptide levels between LAC and normal control groups. The area under the curve (AUC) of the receiver operating characteristic (ROC) curve was used to assessed specificity and sensitivity for each biomarker. Chi-squared tests were used to assess baseline characteristic differences between LAC and control groups and to compare proteins levels in LACs and adjacent normal lung tissues. All tests were two-sided, and *P*<0.05 was considered significant.
